# Range map datasets for terrestrial vertebrates across Taiwan

**DOI:** 10.1016/j.dib.2022.108060

**Published:** 2022-03-16

**Authors:** An-Yu Chang, Wan-Jyun Chen, Rui-Yang He, Da-Li Lin, Yong-Lun Lin, Te-En Lin, Shih-Ping Chou, Chun-Fu Lin, Ruey-Shing Lin, Lin-Wen ChangChien, Shih-Wei Chang, Hsi-Chi Cheng, Yu-Hsiu Lin, Jo-Szu Tsai, Pei-Fen Lee

**Affiliations:** a Endemic Species Research Institute, No.1, Ming-sheng East Road, Jiji, Nantou 552, Taiwan; b Institute of Ecology and Evolutionary Biology, National Taiwan University, No.1, Section 4, Roosevelt Road, Taipei 106, Taiwan; c Wild Bird Society of Hualien, No. 4, Ln. 94, De'an 1st Street, Hualien City, Hualien 970, Taiwan; d School of Biological Science, The University of Queensland, Brisbane, Queensland 4072, Australia; e Department of Biological Resources, National Chiayi University, Chiayi City 60004 Taiwan

**Keywords:** Biodiversity hotspot, Conservation planning, Open datasets, Spatial conservation prioritization, Species distribution models, Spatial geographic range

## Abstract

Accurate data describing the geographic distribution of specific species form the basis for effective conservation management policies. However, for most species the freely available distributional information is usually confined to either expert maps or purely theoretical maps constructed by using a variety of modeling frameworks. These maps usually do not provide enough resolution for conservation applications or do not accurately describe the current distribution status. In this study, we constructed a novel workflow designed to integrate data from various species distribution models and expert knowledge into a single unified modeling process. Under this workflow, we systematically constructed current distribution maps for a selection of terrestrial vertebrates found across Taiwan.

We used species distribution modeling as the base and then aggregated multiple open datasets describing species occurrence and environmental factors as data sources. Thereafter, we estimated the primary broad-scale and high spatial resolution species range maps using the MaxEnt modeling algorithm, and then consulted experts on each taxa to refine these maps.

This dataset provides up-to-date species distribution maps for 379 terrestrial vertebrates in Taiwan, with members from across four taxa (27 amphibians, 52 reptiles, 264 birds, and 36 mammals). This dataset helps to fill the spatial knowledge gaps for conservation concerns and improves our understanding of the geographic distribution of more than half (61%) of the vertebrate species of Taiwan. Furthermore, by stacking the range maps of multiple species, we can identify vertebrate diversity hotspots and identify priority areas for conservation.

## Specifications Table

**Table:** 

Subject	Ecology Nature and Landscape Conservation
Specific subject area	Zoology, Ecology and Conservation Biology
Type of data	Maps, figures, and tables.
How the data were acquired	Both species occurrence and environmental data used in our modeling were collected from open data sources
Data format	Analyzed. Comma separated value tables (.csv) and shapefiles (.shp)
Description of data collection	The species occurrence data were collected from multiple open datasets, including eBird (https://ebird.org/); the ecological database of the Forest Bureau; Taiwan Biodiversity Network (TBN, https://www.tbn.org.tw/); and Global Biodiversity Information Facility (GBIF, https://www.gbif.org). The environmental dataset included multi-temporal and terrestrial environmental data for Taiwan. To ensure that these maps and tables were as current and accurate as possible, we only included data collected from 2010 onwards.
Data source location	Country: Taiwan 21.5° to 26.5° North latitude and between 120° to 122° East longitude.
Data accessibility	Repository name: Mendeley Data Direct URL to data: https://doi.org/10.17632/4g2xfsbmnr.1 Relevant code: https://doi.org/10.5281/zenodo.6257534

## Value of the Data

• The integration of species distribution model (SDM) and expert knowledge facilitates distribution mapping than in traditional expert maps.
• These data fill the knowledge gap in the current spatial distribution of 61% terrestrial vertebrate species in Taiwan.
• The data can be used to assess the threat status of 379 terrestrial vertebrate species by applying the IUCN Red List Categories and Criteria, thereby evaluating the effectiveness of the existing conservation plans and facilitating improved action planning for species conservation.
• This dataset can be used to identify biodiversity hotspots for specific vertebrate species across Taiwan.

## 1 Data Description

**Fig. 1:**
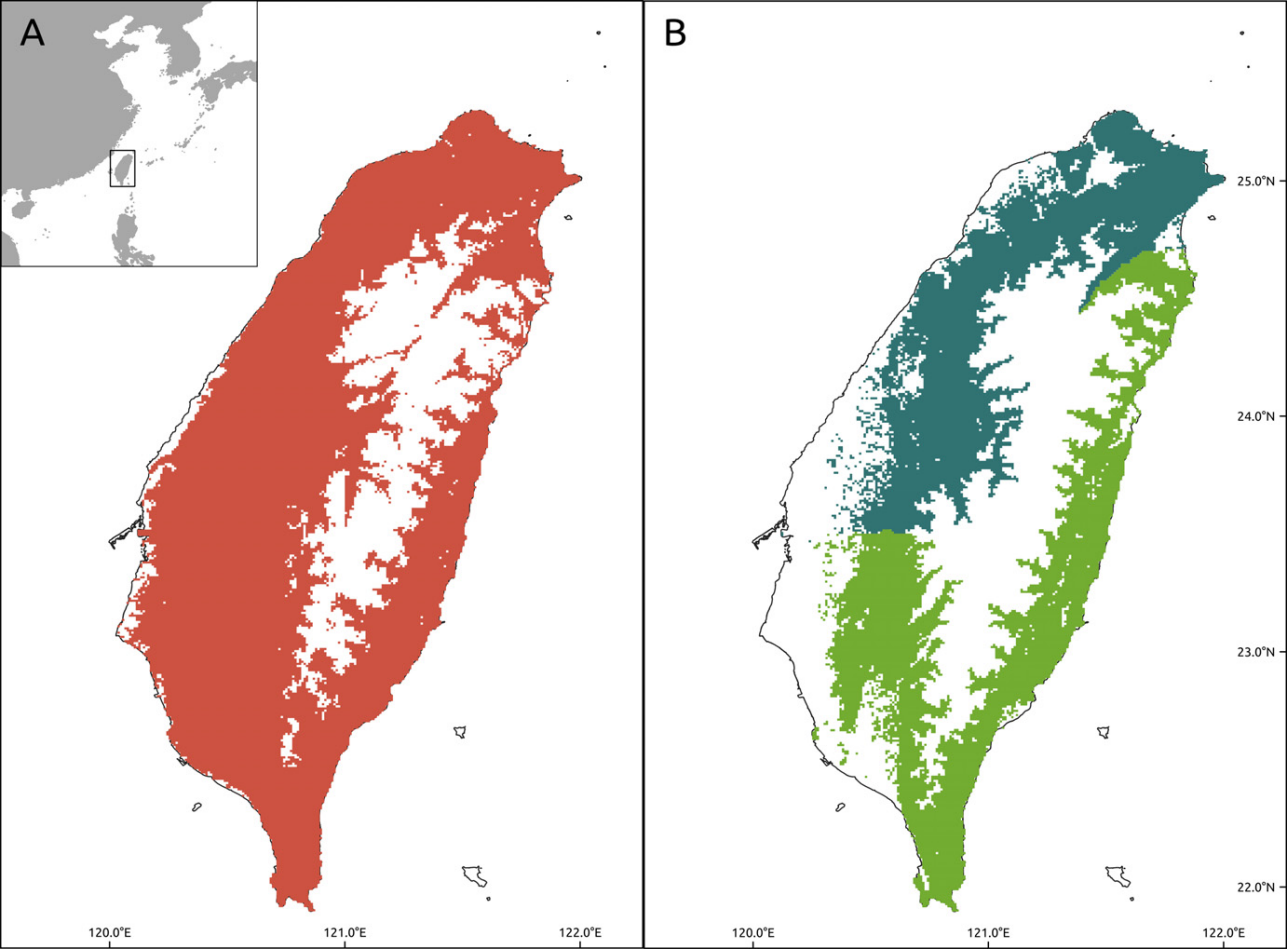
Taiwan, East Asia. (A) Red grids indicate *Diploderma swinhonis* distribution; (B) Range map of *Buergeria choui/otai* species complex–Dark green grids are the area of *B. choui* and light green part presents the distribution of *B. otai*.

This dataset contains the current distribution range maps for 379 terrestrial vertebrates in Taiwan, including their distribution across Taiwan proper (Fig. 1; the outlying islands were excluded). We show the distribution range map of *Diploderma swinhonis*, a common lizard (Fig. 1A), and a complex species range map of *Buergeria choui* and *B. otai* (Fig. 1B) as examples. These maps contain the data for 264 birds, 36 mammals, 52 reptiles, and 27 amphibians (Table 1) and the metadata for each range map. The metadata table includes the detailed information such as species name, occurrence sample size, modeling results, and reviewers of each map (Appendix 1).

**Table 1:** Number of native terrestrial vertebrate species in Taiwan proper and species with validated distribution models constructed in this study.

Taxon	Number of Species in Taiwan [2]	Number of Species with Validated Model Data
Amphibia	38	27 (71%)
Reptilia	80	52 (65%)
Aves	418*	264 (63%)*
Mammalia	83	36 (43%)

⁎ not including vagrant species and pelagic birds.

These distribution range maps were constructed using the occurrence data from 2010 to 2020. In addition, we also used the environmental variables in the 2010s to simulate the distribution model (Table 2) and ensure species relevance as well as the environment characteristics. These data were then used to determine the suitable habitat for each species as determined using MaxEnt software (version 3.4.1) [1]. Then, each habitat map was refined and validated by taxonomic experts to generate our primary species distribution maps.

**Table 2:** Environment variables included when constructing the species distribution models.

Variable Class	Variable Name	Unit	Description
Climate	Bio1	°C	Annual mean temperature
Climate	Bio2	°C	Mean diurnal range
Climate	Bio4	°C	Temperature seasonality
Climate	Bio12	mm	Annual precipitation
Climate	Bio15	%	Precipitation seasonality
Climate	Bio16	mm	Precipitation in the wettest quarter
Topographic	Aspect	degree	-
Topographic	ASR	kWh/m^2^	Area of solar radiation
Topographic	ELE	meter	Mean elevation
Topographic	ELESD	meter	Standard deviation of elevation
Topographic	Slope	degree	-
Land cover	BL	m^2^	Area of bare land
Land cover	BU	m^2^	Area of bush
Land cover	FF	m^2^	Area of farmed fields
Land cover	FO	m^2^	Area of forest
Land cover	FW	m^2^	Area of farmed wetland
Land cover	MD	m^2^	Area of meadow
Land cover	UB	m^2^	Area of urban development
Land cover	WB	m^2^	Area of waterbody
Land cover	WL	m^2^	Area of wetland
Others	DFW	meter	Distance to the nearest body of freshwater

All data are available from both Mendeley Data and the Taiwan Biodiversity Network (TBN) [2].

## 2 Experimental Design, Materials and Methods

**Fig. 2:**
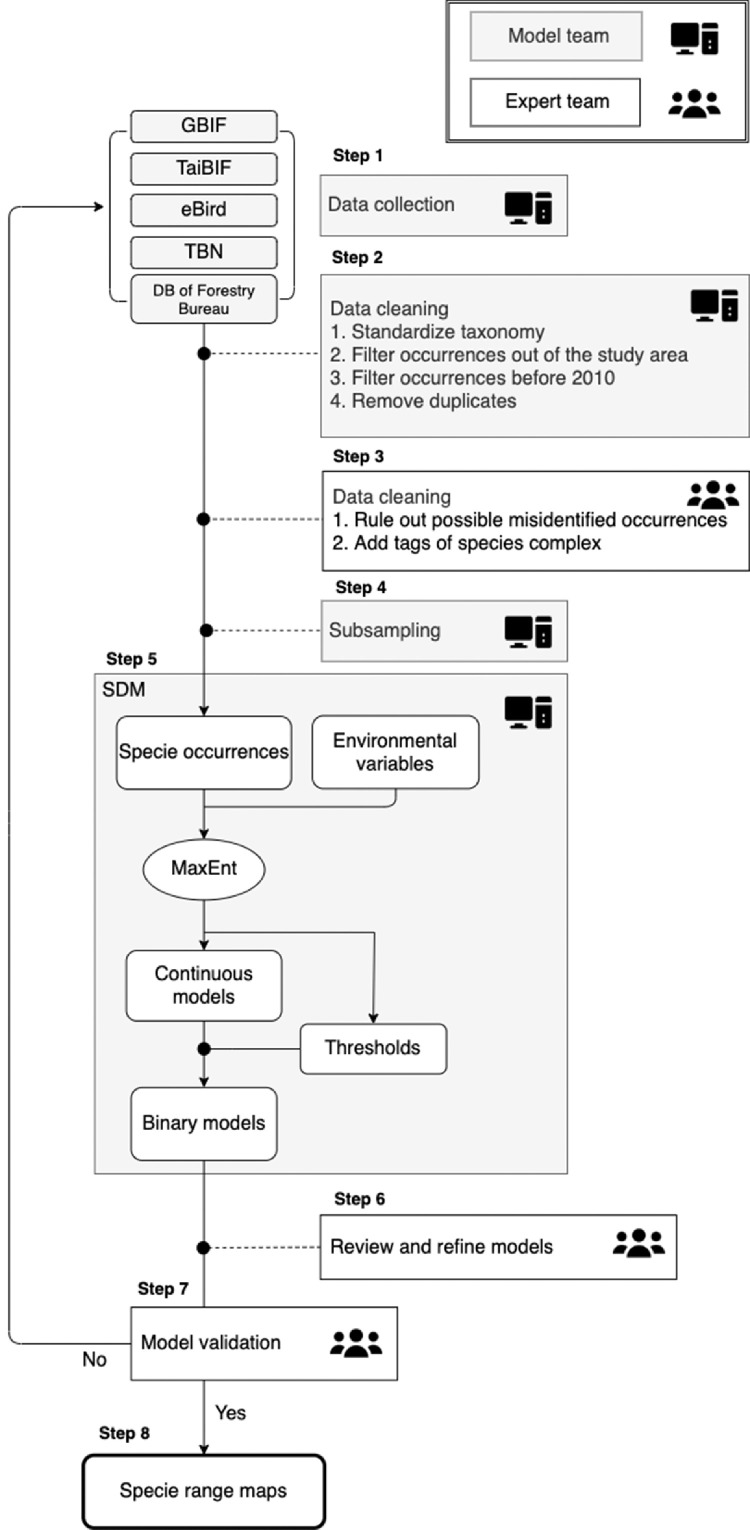
Evaluation of the framework used for generating the new species range maps described in this dataset. Step 1, species occurrence data collection from open datasets; Step 2, logical data cleaning, including standardizing the taxonomy, filtering occurrence data outside the study area and before 2010, and removing spatial duplicates; Step 3, data cleaning based on expert opinions, checking misidentified species and tagging the species complex; Step 4, spatial subsampling of single species occurrence data into a 5 km resolution; Step 5, species distribution model (SDM) construction by species occurrence data and environmental variables, in which the MaxEnt software provided 11 types of thresholds to binary species distribution maps from the continuous model results; Step 6, reviewing and refining of the binary species distribution maps by taxonomic experts, who then select a representative map; Step 7, expert validation of the model data; and Step 8, exporting of the species range maps. If the species model result was not accepted at Step 6, the work did loop back to start again from Step 1 to collect additional occurrence data.

We constructed a novel framework for the analysis of occurrence and environmental data to facilitate improved modeling of terrestrial vertebrate distributions in Taiwan. This model used the loop information from the occurrence raw data and machine learning model to fit the data with expert opinions to construct reliable, reproducible range maps. This system integrates data from multiple data sources, data cleaning, modeling, and expert review to produce superior modeled datasets. All data processing and analysis were performed using R version 3.5.3 [3] and QGIS version 3.4.6 [4] and are summarized in Fig. 2. The R script is available at GitHub (https://github.com/kemushi54/Range-map-datasets-for-terrestrial-vertebrates-across-Taiwan; 10.5281/zenodo.6257534).

### 2.1 Occurrence data preparation

We integrated terrestrial vertebrate species occurrence data from multiple open data sources and portals, including eBird [5], the ecological database for the Forest Bureau [6], TBN, and the Global Biodiversity Information Facility (GBIF, [7]), and performed a series of data quality checks on each dataset, including standardizing the taxonomy in each dataset to conform to the TBN taxonomy tree [2], filtering out the data from outside of our study area, and removing any spatial duplicates (i.e., keep only one record per one 1 km cell). These occurrence data were then reviewed by experts who identified and removed any likely misidentifications species by species before the data were used in the SDM construction process. These experts also added species complex information tags at this step. We then aggregated the occurrence data by species complex tag. We limited the occurrence data evaluated in this study to those produced after 2010.

Spatial bias and clustering in the occurrence data are frequent phenomena, especially for data aggregated from multiple sources, crowdsourced data, and random sampling of citizen science data [8], [9], [10]. As this issue needs to be addressed to meet the baseline assumptions used in MaxEnt [8,11], we implemented a spatial subsampling step for each of the species/species complex groups before model estimation. This was completed by dividing our sampling region into 5 × 5 km cells and then selecting a single occurrence within each cell for evaluation. Where there was more than one occurrence within a single cell, we randomly chose one set of data for evaluation. We generated 20 subsampled datasets for each species/species complex and only constructed models of species/species complexes with more than 30 occurrences after spatial subsampling.

### 2.2 Environmental data preparation

We used environmental variables from a multi-temporal, terrestrial environment dataset from Taiwan [12]. This environmental dataset contains data gathered in several decades for different categories of environmental variables (climatic, bioclimatic, topographic, land cover, etc.). The spatial resolution of this dataset is 1 km, and the data type was continuous quantitative. We also restricted the environmental data to the 2010s to combine the occurrence and environmental data in our models.

We avoided collinearity by excluding any variables with a high degree of correlation (|r| > 0.75) and were left with 21 environmental variables for our modeling exercise, namely, six bioclimatic, five topographic, nine land cover, and one “other” variable (Table 2).

### 2.3 Modeling

The distribution models were generated using MaxEnt software (version 3.4.1). The MaxEnt method was selected because it requires only presence records, and it performs well even if there are only a few records for evaluation [13]. As we constructed several models for multiple species simultaneously, we used the default software settings: cloglog transform output, no bias-files, and 10,000 background points, with a regularization multiplier of one [14].

We ran five replicates for each subsampling dataset for each species/species complex and retained 20% of each of these to use as random test data (i.e., 100 sub-models for each species/species complex). Thus, the averages of the suitability values from each of these 100 individual sub-models were viewed as one model prediction.

### 2.4 From continuous model to distribution range

Given that the quality of the occurrence data differs across species, we generated binary species distributions based on individual species-specific thresholds. For each individual model, MaxEnt provides 11 types of thresholds calculated using different approaches. We averaged these 11 threshold values from the 100 sub-models for each species/species complex and used these to convert our continuous models into 11 binary models. Then, the taxonomic experts selected an appropriate binary model for each species/species complex that best represented the optimal environmental conditions.

All models were reviewed and refined by the experts who removed areas of model overestimation. In addition, range maps for each of the individual species tagged as part of the same species complex were constructed separately at this step by the experts.

For example, *Buergeria choui* and *B. otai* are tagged as the same species complex (*Buergeria choui/otai* complex) because they are morphologically very similar, use almost the same habitat, and were only separated as two different species in 2020. The occurrence of these two species was then reidentified as one species complex (Step 3 in Fig. 2) and treated as one species during the model construction process (Steps 4–5 in Fig. 2). Then, the experts generated individual species’ range maps (Fig. 1B) for each of these vertebrates using the species complex distribution model. These two species were then shown to be geographically isolated; thus, the experts were able to draw the new range distribution maps by using their known geographic boundaries.

### 2.5 Model evaluation and validation

We then evaluated the quality of each model for the individual species/species complex data by using an average of the AUC_training_ and AUC_diff_ values from each of the 100 sub-models. In addition, for every species/species complex, we asked at least one additional expert to validate the refined binary models. As a result, each model was flagged as validated or not recommended for use. Only the maps flagged as validated were included in our published dataset, which includes species distribution maps for 379 terrestrial vertebrates in Taiwan, with members from across four taxa (27 amphibians, 52 reptiles, 264 birds, and 36 mammals). This dataset could be particularly useful for conservation planning and evaluating the effectiveness of the existing conservation plans.

## Ethics Statements

Our work did not involve human subjects or animals. We collected data from open datasets, excluding social media platforms.

## CRediT authorship contribution statement

**An-Yu Chang:** Conceptualization, Methodology, Software, Writing – original draft, Visualization. **Wan-Jyun Chen:** Conceptualization, Methodology, Writing – original draft, Writing – review & editing, Supervision. **Rui-Yang He:** Validation. **Da-Li Lin:** Validation. **Yong-Lun Lin:** Validation. **Te-En Lin:** Resources, Validation. **Shih-Ping Chou:** Validation. **Chun-Fu Lin:** Validation. **Ruey-Shing Lin:** Validation, Writing – review & editing. **Lin-Wen ChangChien:** Validation. **Shih-Wei Chang:** Validation. **Hsi-Chi Cheng:** Resources, Validation. **Yu-Hsiu Lin:** Resources, Validation. **Jo-Szu Tsai:** Resources, Validation, Writing – review & editing. **Pei-Fen Lee:** Writing – review & editing.

## Declaration of Competing Interest

The authors declare that they have no known competing financial interests or personal relationships that could have appeared to influence the work reported in this paper.

## Data Availability

Range map dataset for terrestrial vertebrates across Taiwan (Original data) (Mendeley Data).

## Appendix Supplementary materials

Image, application 1

## Footnotes

Supplementary material associated with this article can be found in the online version at doi:10.1016/j.dib.2022.108060.
